# Developments in FTICR-MS and Its Potential for Body Fluid Signatures

**DOI:** 10.3390/ijms161126012

**Published:** 2015-11-13

**Authors:** Simone Nicolardi, Bogdan Bogdanov, André M. Deelder, Magnus Palmblad, Yuri E. M. van der Burgt

**Affiliations:** 1 Center for Proteomics and Metabolomics, Leiden University Medical Center (LUMC), PO Box 9600, 2300 RC Leiden, The Netherlands; s.nicolardi@lumc.nl (S.N.); a.m.deelder@lumc.nl (A.M.D.); n.m.palmblad@lumc.nl (M.P.); 2 PerkinElmer, San Jose Technology Center, San Jose, CA 95134, USA; bogdan.bogdanov@perkinelmer.com

**Keywords:** mass spectrometry, proteomics, solid-phase extraction, ultra-high resolving power, FTICR-MS, instrumental development, serum, saliva, clinical cohort, profiling

## Abstract

Fourier transform mass spectrometry (FTMS) is the method of choice for measurements that require ultra-high resolution. The establishment of Fourier transform ion cyclotron resonance (FTICR) MS, the availability of biomolecular ionization techniques and the introduction of the Orbitrap™ mass spectrometer have widened the number of FTMS-applications enormously. One recent example involves clinical proteomics using FTICR-MS to discover and validate protein biomarker signatures in body fluids such as serum or plasma. These biological samples are highly complex in terms of the type and number of components, their concentration range, and the structural identity of each species, and thus require extensive sample cleanup and chromatographic separation procedures. Clearly, such an elaborate and multi-step sample preparation process hampers high-throughput analysis of large clinical cohorts. A final MS read-out at ultra-high resolution enables the analysis of a more complex sample and can thus simplify upfront fractionations. To this end, FTICR-MS offers superior ultra-high resolving power with accurate and precise mass-to-charge ratio (*m*/*z*) measurement of a high number of peptides and small proteins (up to 20 kDa) at isotopic resolution over a wide mass range, and furthermore includes a wide variety of fragmentation strategies to characterize protein sequence and structure, including post-translational modifications (PTMs). In our laboratory, we have successfully applied FTICR “next-generation” peptide profiles with the purpose of cancer disease classifications. Here we will review a number of developments and innovations in FTICR-MS that have resulted in robust and routine procedures aiming for ultra-high resolution signatures of clinical samples, exemplified with state-of-the-art examples for serum and saliva.

## 1. Introduction

Fourier transform ion cyclotron resonance (FTICR) mass spectrometry (MS) offers the highest resolving power, resolution, and mass-to-charge ratio (*m*/*z*) measurement accuracy of all existing MS techniques [1]. This unparalleled performance has been heavily challenged since the commercial introduction of the Orbitrap™ mass spectrometer, the newest member of the FTMS family [2]. Nevertheless, the Orbitrap’s inventor himself stated that “inherent stability and field uniformity of superconducting magnets in synergy with the very high accuracy and dynamic range of frequency measurements made this technique an ultimate champion in mass resolving power and mass accuracy” [3]. The isotopic distribution accuracy, a wide intra- and inter-spectrum dynamic range, and high sensitivity all make FTICR-MS the technique of choice to analyze samples of high complexity and identify thousands of compounds simultaneously with high confidence, with petroleomics and analysis of complex natural products being key examples [4,5,6]. In addition, FTICR plays its role in bottom-up and top-down proteomics approaches, and recently also in middle-down applications [7,8,9]. Since its introduction forty years ago the basic design has not changed: internally or externally generated ions are radially trapped in a strong magnetic field combined with a weak axial electric field, the image current is being detected from the coherently excited trapped ions, and the resulting time-domain signal is digitized and converted using Fourier transform (FT) into the frequency-domain spectrum and finally into a mass spectrum [10]. In addition, FTICR-MS has undergone many technological improvements of its various components, both with regard to hardware and software. Improved resolving power, resolution, *m*/*z*-accuracy, dynamic range, and sensitivity of FTICR-MS are not the result of the development of a single instrument component. Modern FTICR instruments are truly wonders of science and engineering. For the main portion of its lifetime FTICR-MS has been the domain of the academic research environment, but nowadays commercial instruments are available to a larger and less specialized group of users, mainly because of the development of easy-to-use control software which required less or no instrument tuning and the possibility to set up complex experiments. This development has also opened the door towards large-scale clinical applications. Here we will overview these instrumental improvements and present two different examples of body fluid signatures obtained with FTICR-MS, namely peptides and small proteins from serum and peptides from saliva.

## 2. FTICR-MS Instrumental Developments

### 2.1. FTICR-MS Components

Most FTICR-MS applications are based on electrospray ionization (ESI) or other atmospheric pressure ionization techniques to generate and introduce ions into the instrument [11]. Studies that use matrix-assisted laser desorption/ionization (MALDI) have been reported less frequently despite the excellent fit of MALDI and FTICR-MS [12,13,14,15,16]. In both cases ions enter the instrument after generation at atmospheric pressure and make their way towards the ultra-high vacuum (UHV) region and into the ICR cell that resides inside the homogeneous region of a high-field superconducting magnet. Improved inlet capillaries and the use of the ion funnel have reduced the ion losses dramatically by making the first stage of the ion transfer more efficient [17]. New ion funnel designs like the dual ion funnel, the high-pressure ion funnel, and the ion funnel trap have been or will be able to improve FTICR-MS even more when implemented [18,19,20]. Implementation of the RF/DC selection quadrupole and the linear ion trap have contributed to the increase in the dynamic range, in addition to providing more selective tandem mass spectrometry (MS/MS) capabilities [21,22]. The dynamic range enhancement applied to mass spectrometry (DREAMS) technology developed at the Pacific Northwest National Laboratory (PNNL) by Smith and co-workers improves the detectability of lower ion count peptides among high ion count co-eluting peptides, but unfortunately never has been commercialized even though it was implemented on an LTQ-FT instrument with automatic gain control (AGC) capabilities [23]. After ion trapping in a multipole ion guide/trap, the ions have to be ejected towards the ICR cell for the second trapping event and angled extraction wires have made this process more efficient [24]. Even though a field-free flight tube has been implemented, the use of a multipole ion guide offers a more efficient option to accomplish the ion transfer to the ICR cell. However, time-of-flight effects during the ion transfer might lead to skewed mass spectrum appearances [25]. In order to reduce these effects, solutions like the restrained ion population transfer, a shorter ion guide, multiple ion filling and transfer times, and the hexapole and octopole ion guides have been implemented [25,26,27,28]. In addition, various effects when the ions enter the magnetic field have been reported and accounted for [29,30]. Finally, advances in data acquisition, storage, processing, and analysis have been detrimental. Calibration taking into account ion population dependency, the “walking” calibration, and artificial neural network calibration have made sub-ppb mass accuracy possible [31,32,33]. In addition, centroid data storage, absorption mode/phase correction, apodization/zero-filling, double/multiple frequency measurements, and perhaps in the future the filter diagonalization method (FDM) or other alternatives instead of FT have or will make FTICR faster and perform better [34,35,36,37].

### 2.2. ICR Cell Ion Trapping and MS/MS

Initially, gas-assisted ion trapping was needed during the ICR cell ion trapping event. This prevented high-throughput applications of FTICR-MS because of the low-duty cycle due to the time required to remove trapping gas to obtain UHV essential for improved FTICR-MS performance. After the implementation of an extra external trapping event new ICR trapping methods like side-kick and gated trapping were introduced and the gas pulse was no longer needed [38,39,40]. FTICR-MS performance is very sensitive to the ion population inside the ICR cell [41]. Too many ions in the ICR cell will lead to space charge effects and frequency shifts might occur, which could influence the resolving power, the dynamic range, the *m*/*z-*accuracy, and the peak shapes [42]. The introduction of AGC, which was commonly used for the same reasons on quadrupole ion trap instruments, for FTICR-MS was a big step towards tackling these challenges [22]. Once inside, the ICR cell the ions can undergo various events like an additional high resolution ion isolation, for both single or multiple ions by CHEF or SWIFT or the multiplexed analogues, respectively, ion-molecule reactions like HDX, or MS/MS by sustained off-resonance irradiation—collision-induced dissociation (SORI-CID), electron capture dissociation (ECD), infrared multi-photon dissociation (IRMPD), ultraviolet photo-dissociation (UVPD), or surface-induced dissociation (SID) [43,44,45,46,47,48,49,50]. Ion excitation has been performed by applying CHIRP or SWIFT waveforms and the excitation radius has a noticeable influence on the FTICR merits of performance [51,52,53]. In addition, the transient lifetime could be extended by applying electron promoted ion coherence (EPIC), and 60° excitation plates (and consequently 120° detection plates) showed the expected improved performance [25,54]. Improved designs of the pre-amplifier and the ICR cell UHV by using turbo pumps with magnetic field shielding have made their positive contributions as well [55,56].

### 2.3. ICR Cell Improvements

The increase in the magnetic field strength (B) and its homogeneity has been very important to the performance improvements. Until very recently, the highest magnetic field strength with a superconducting wide bore magnet was 15 tesla. Since then, two FTICR-MS systems equipped with 21 tesla superconducting magnets have been installed at the National High Magnetic Field Laboratory (NHMFL) and at PNNL [57,58]. The increase in the magnetic field strength improves the resolving power, the highest non-coalesced mass, the duty cycle (all proportional to B), and the trapped-ion upper mass, the ion trapping time, the ion energy, and the number ions (all proportional to B^2^) [59]. The first publicly shared results of the NHMFL 21 tesla FTICR showed the expected and impressive results [57]. The ICR cell is the other key element that has attracted a lot of attention to improve upon and over the past forty years many different designs have been developed [59]. The original cubic ICR cell has been succeeded by mainly cylindrical-shaped designs, although a hyperbolic design has been reported [60,61]. Only the matrix-shimmed cube design was a reminder of how it all once started [62]. The cylindrical designs are either open (with four trapping plates on each side of the two excitation and detection plates, and either uncoupled or coupled trapping and excitation plates), or closed (with two cylindrical trapping plates in the *xy* plane) [63,64]. The “infinity” cell is a version of the closed cylindrical ICR cell [39]. In addition, various dual-cell designs have been reported [65,66]. Over the past decade a relatively large number of innovative ICR cells have been designed and constructed and the main goal of all designs was to increase the resolving power by extending the transient lifetime and to decrease the space-charge effects and resulting frequency shifts to maintain a high *m*/*z*-measurement accuracy without compromising peak shapes through peak splitting or peak coalescence. For more detailed descriptions and motivations, we refer the reader to the cited references [57,58,59,60,61,62,63,64,65,66]. The complexity of the ion motion inside an ICR cell and the interactions of the three motion components, the cyclotron rotation, the trapping oscillation, and the magnetron rotation, all with their own *m*/*z*-dependent frequency, have led to various approaches and resulting ICR cell designs [59]. The trapping ring electrode cell, the electrically compensated cell, the grid/Ultra cell, the multipole cell, the open cylindrical seven segment cell [67], the temperature-controlled cell, the coaxial multi-electrode cell/O-trap, the dynamically harmonized cell, and the narrow aperture detection electrodes cell all have shown the targeted improvements on the FTICR-MS performance [53,67,68,69,70,71,72,73,74]. One of the most recent additions, the dynamically harmonized cell, has been extensively characterized theoretically and several new resolving power records have been accomplished [41,75,76]. Initially, using a 7 tesla FTICR-MS instrument for protonated reserpine a resolving power of 24,000,000 was obtained using a 180 s time domain signal, and for the 49+ charge state of bovine serum albumin (BSA) a resolving power of 1,200,000 was obtained [73]. Next, using a different 7 tesla FTICR-MS instrument and applying phase correction (the absorption-mode) for protonated reserpine, a resolving power of 32,000,000 was obtained [77]. Minor frequency shifts were observed when changing the excitation radius and accurate isotope fine structure was determined for the peak distributions of reserpine and Substance P, hereby confirming the elemental compositions [78]. In addition, the dynamically harmonized cell is commercially available on the Bruker Daltonics SolariX XR™ FTICR (Bremen, Germany) instrument so a larger audience can have access to ultra-high resolution MS capabilities and their benefits to solve scientific questions that other MS technologies cannot [79].

## 3. State-of-the-Art FTICR-MS Body Fluid Peptide and Protein Signatures

In this part of the review we present two examples of body fluid signatures obtained on a 15 tesla FTICR-MS instrument equipped with an ESI/MALDI ion source and an implemented dynamically harmonized cell. For most practical proteomics and metabolomics ESI-based applications resolving powers of 100,000–200,000 are sufficient, which are values that can only be obtained by FTICR and Orbitrap™ instruments and currently not by any commercially available TOF instrument. Previously, we have reported various high-throughput solutions for balancing the speed of the current FTICR-MS-acquisitions (“measurements”) and the time needed for analytical cleanup of body fluids (“sample preparation”) [13,80,81,82]. In this context it is stressed that time-consuming sample preparation procedures such long-gradient bottom-up liquid chromatography (LC)-MS/MS proteomics workflows are impracticable for high-throughput analyses. Moreover, such approaches require specific optimization to allow a robust and reproducible throughput of complex biological samples. A robotic pipetting platform for single-step sample cleanup and separation of complex protein mixtures is extremely helpful in providing a high degree of standardization. With proper calibration and maintenance of the liquid handling system on a regular basis, all procedures are performed in exactly the same way and as a result the precision improves of the sample preparation is improved [82,83,84]. Furthermore, sample processing times can be decreased and automation enables 24/7 performance.

### 3.1. Example 1: MALDI-FTICR-MS Profiling of Serum Peptides and Proteins

**Figure 1:**
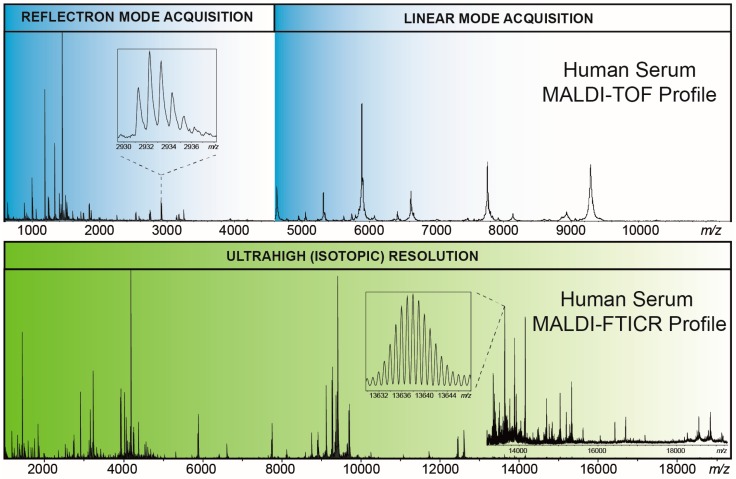
Early (in blue) and current (in green) matrix-assisted laser desorption/ionization (MALDI)-based serum peptide and protein profiles obtained after reversed-phase (RP) C18-magnetic bead cleanup. The MALDI-time-of-flight (TOF) profile in the upper panel is composed of a low-resolution linear mode spectrum (up to *m*/*z*-value 11,000) and a high-resolution reflectron mode spectrum (up to *m*/*z*-value 4600); in the lower panel a MALDI- Fourier transform ion cyclotron resonance (FTICR) profile of the same sample is shown.

Proteins and peptides were isolated (fractionated) from human serum samples using functionalized paramagnetic beads purchased from Invitrogen (reversed-phase (RP) C18 Dynabeads; Carlsbad, CA, USA) as described previously [80,85]. Briefly, a single-step serum cleanup procedure based on solid-phase extraction (SPE) was combined with MALDI-FTICR readout. Fresh RPC18-functionalized magnetic beads were first activated by a three-step washing protocol on a fully automated 96-channel liquid handling system. Then, the serum samples were added and incubated, followed by washing the beads and eluting the peptides with a 1:1 mixture of water and acetonitrile. Spotting onto a MALDI AnchorChip™ (Bruker Daltonics, Bremen, Germany) target plate was performed in combination with a mixing protocol using MALDI matrix α-cyano-4-hydroxycinnamic acid (CHCA, Bruker Daltonics, Bremen, Germany). MALDI-FTICR profiles were obtained on a Bruker 15 tesla SolariX XR™ FTICR mass spectrometer equipped with an ESI/MALDI CombiSource™, a novel ParaCell™ and a Bruker Smartbeam-II™ Laser System. Two acquisition settings (a low-mass (LM) and high-mass (HM) method) were used to optimize both the sensitivity and the resolving power in the range from 1012 to 5000 *m*/*z*-units and from 3497 to 30,000 *m*/*z*-units, respectively. A typical example of a compiled MALDI-FTICR “RPC18-profile” of a human serum sample is presented in Figure 1. For comparison, a MALDI-time-of-flight (TOF) profile of the same sample is shown in combination with this ultra-high resolution profile. MALDI-TOF is by far the most widely used strategy in MS-based biomarker profiling studies [86,87,88,89,90,91]. Unfortunately, amongst other issues, the identification of discriminating features (*i.e.*, peptides) in such MALDI-TOF profiles has been rather unsuccessful. Here, two beneficial aspects of ultra-high resolution of FTICR come into play: all species observed in Figure 1 were isotopically resolved up to 19,000 mass units thus facilitating their identification, and overlapping signals can be resolved. Furthermore, it follows from Figure 1 that the relative intensities of all species do not simply overlay between TOF and FTICR. This illustrates that the signatures are platform-specific and stresses the need for calibrators for quantification purposes [83].

### 3.2. Example 2: MALDI-FTICR-MS Profiling of Salivary Peptides and Proteins

**Figure 2:**
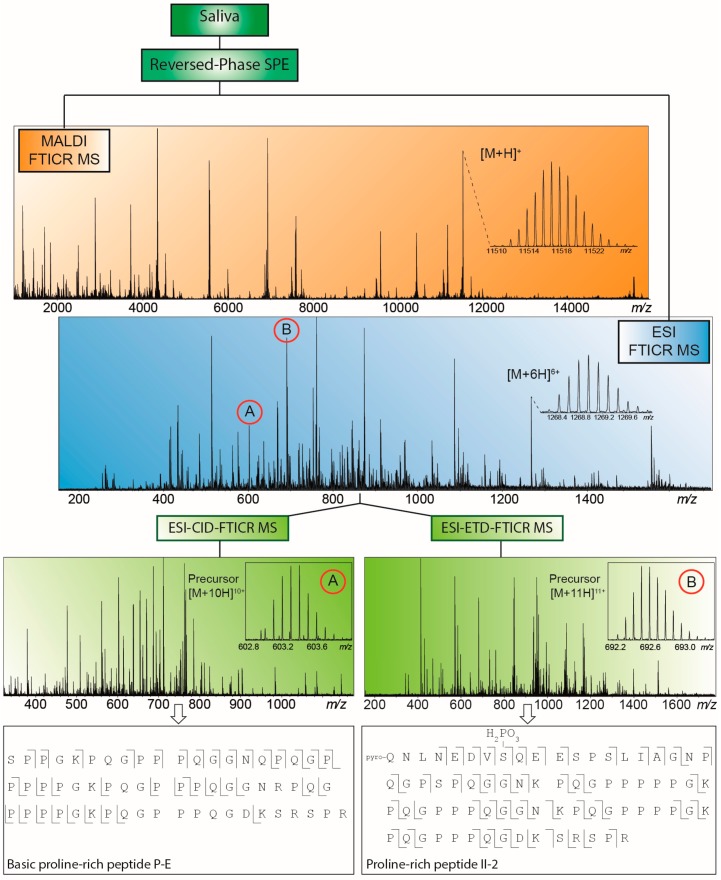
MALDI-FTICR (in orange) and electrospray ionization (ESI)-FTICR (in blue) saliva peptide and protein profiles obtained after RPC18-based cleanup. Multiply charged species in the ESI-FTICR profile (indicated with A and B) were selected for tandem mass spectrometry (MS/MS) analysis, with identification results shown at the bottom (in green).

As a second example a typical MALDI-FTICR “RPC18-profile” of a human saliva sample is presented in Figure 2. Note that all species observed in Figure 2 were isotopically resolved up to 15,000 mass units. The ultra-high resolution of FTICR enables structure characterizations of (interesting) features in a profile. This is even more facilitated in the ESI-mode when on the same system a profile is obtained with multiply charged species. These are convenient for identification purposes, as is demonstrated at the bottom of Figure 2. Two large peptides were identified from a CID- and an ETD-MS/MS spectrum, respectively.

Provided the MALDI-based profiles can be obtained in a robust way, that is, at minimum considering the basic analytical quality requirements, these peptide and protein signatures have potential for clinical applications [83]. Topics such as costs of the MS-system and “return-on-investment” obviously become important when aiming for future routine measurements and implementation as an assay. Clearly, FTICR platforms are expensive and operating costs are higher than for an Orbitrap™ system due to the need for liquid helium. However, FTICR magnets last long (more than 15 years is not uncommon) and instrument down-time is low. Consequently, it may well be that the comparison with other routine, often also expensive clinical assays is beneficial for FTICR. Furthermore, FTICR data storage is no longer a limiting factor and current systems allow future re-analysis, which is advantageous for longitudinal studies.

## 4. Conclusions and Future Outlook

The most recent promising improvements in FTICR hardware and software have been made at universities and national labs. With only one FTICR manufacturer left and the reality of patents and licenses, it might be some time before some of these will be available on commercial instruments. However, the current FTICR instruments have many more advanced technologies compared to a decade ago and are suitable for the kind of high throughput clinical screening applications described here. As was shown in this review, peptide and protein signatures can be obtained via a standardized and automated one-step sample cleanup strategy in combination with high-end MS readout using ultra-high resolution equipment. These signatures can be classified and provide a solid platform for rapid screening of body fluids in order to further select a sub-set for detailed (or in-depth) analysis. These workflows can also be applied for the analysis of lipids, and glycans and glycopeptides derived from glycoproteins [84]. Hyphenation of ion mobility, using either high-field asymmetric waveform ion mobility mass spectrometry (FAIMS) or drift tubes, and FTICR will open new opportunities to increase the dynamic range and the selectivity. It will be interesting to see if the *m*/*z*-range of FTICR can be further extended (to for instance *m*/*z*-values of 25,000 or even up to 50,000) so more proteins can be detected and to follow developments in Orbitrap™ technology. This will not only be of interest for MALDI-based profiling studies, but also for tissue-based MALDI-MS imaging research [92,93]. It is further noted that the application of direct infusion native nano-ESI in (clinical) protein screening or analysis of biopharmaceuticals might gain interest. For example, protein complexes and viruses have been analyzed on an optimized Orbitrap™ platform [94,95]. Finally, sub-ppm mass precision and accuracy allow determination of isotopic distributions and exact mass measurements and open the way for isotopic fine structure analyses for identification purposes [78,96].
